# Ochratoxin A Sequentially Activates Autophagy and the Ubiquitin-Proteasome System

**DOI:** 10.3390/toxins11110615

**Published:** 2019-10-24

**Authors:** Hafize Aysin Akpinar, Hilal Kahraman, Ibrahim Yaman

**Affiliations:** 1 Molecular Toxicology and Cancer Research Laboratory, Department of Molecular Biology and Genetics, Bogazici University, Bebek-Istanbul 34342, Turkey; aysindemirkol@gmail.com (H.A.A.); hilalkahramann@gmail.com (H.K.); 2 Center for Life Sciences and Technologies, Bogazici University, Bebek-Istanbul 34342, Turkey

**Keywords:** ochratoxin A, ubiquitin-proteasome system, autophagy, proteolytic pathways, PI3K/AKT, MAPK/ERK1-2

## Abstract

Ochratoxin A (OTA) is a carcinogenic mycotoxin, which is produced by *Aspergillus* and *Penicillium* genera of fungi and commonly contaminates food and feed. We and others have previously shown that OTA causes sustained activation of PI3K/AKT and MAPK/ERK1-2 signaling pathways in different cell types and animal models. Given the close relationship between cellular signaling activity and protein stability, we were curious whether increased PI3K/AKT and MAPK/ERK1-2 signaling may be the result of OTA-stimulated alterations in proteolytic activity. We show that both of the major proteolytic systems, autophagy, and the ubiquitin-proteasome system (UPS), are activated upon OTA exposure in human kidney proximal tubule HK-2 and mouse embryonic fibroblast (MEF) cells. OTA stimulates transient autophagic activity at early time points of treatment but autophagic activity subsides after 6 h even in the sustained presence of OTA. Interestingly, OTA exposure also results in increased cell death in wild-type MEF cells but not in autophagy-halted *Atg5*-deficient cells, suggesting that autophagy exerts a pro-death effect on OTA-induced cytotoxicity. In addition, prolonged OTA exposure decreased ubiquitinated protein levels by increasing proteasomal activity. Using purified and cellular proteasomes, we observed enhanced chymotrypsin-, caspase-, and trypsin-like activities of the 26S but not the 20S proteasome in the presence of OTA. However, in the cellular context, increased proteasomal activity depended on prior induction of autophagy. Our results suggest that autophagy and subsequent UPS activation are responsible for sustained activation of PI3K/AKT and MAPK/ERK1-2 pathways through regulating the levels of critical phosphatases VHR/DUSP3, DUSP4, and PHLPP, which are known to be involved in OTA toxicity and carcinogenicity.

## 1. Introduction

Ochratoxin A (OTA) is a secondary metabolite of some species of *Aspergillus* and *Penicillium* genera of fungi [1], which can contaminate a number of foods and feed. Since the whole intact molecule is permeable to cell membranes [2], OTA exposure and uptake by humans and animals are relatively high compared to other contaminants. Potential negative health effects of OTA are further increased by its long half-life, which is approximately 35 days in the human circulation after a single dose of exposure [3,4]. Consequently, immunotoxic, neurotoxic, hepatotoxic, teratogenic, and potentially carcinogenic effects of OTA have been demonstrated in rodent systems [5]. However, the mode of action of OTA is not fully understood at the molecular level. OTA has been proposed to exert genotoxic effects by forming DNA adducts [6] but has also been implicated in other processes, such as epigenetic changes through deregulation of signaling pathways [7], oxidative DNA damage via increased reactive oxygen species (ROS) release, and suppression of apoptosis and cell cycle progression [8,9].

It has previously been shown that OTA causes deregulation of several signaling pathways associated with cell death and survival in a time- and dose-dependent manner [10]. Even though, the genotoxic and non-genotoxic effects of OTA were investigated in some detail, data describing its effect on cellular proteolytic pathways, which among other functions could affect signaling activity in cells, are scarce [11]. Generally, modified, tagged, and misfolded proteins may undergo lysosomal and/or proteasomal degradation [12,13], which is important for cellular homeostasis, including proper protein metabolism and regulation of the signaling pathways. 

Autophagy is a cellular process that includes the digestion of cellular contents in lysosomes and three major types of autophagy have been defined; (i) macroautophagy, (ii) microautophagy, and (iii) chaperon-mediated autophagy [14]. Macroautophagy, referred to as autophagy hereafter, is defined as the degradation of macromolecules and organelles during which substrates are enclosed by the double-layered membrane of the autophagosome and carried to the lysosomes for digestion. 

The ubiquitin-proteasome system (UPS) is the second major protein degradation pathway in eukaryotic cells and is responsible for the degradation of damaged, misfolded, and short-lived proteins. Two main entities function in the UPS in a coordinated manner; (i) the E1, E2, and E3 enzymes, which ubiquitinate target protein, and (ii) the proteasome itself, which is responsible for the degradation of ubiquitinated proteins [15]. The proteasome is a large multienzyme complex that is composed of two major subunits and containing three distinct catalytic sites with caspase-, chymotrypsin-, and trypsin-like proteolytic activities [16].

Although, all types of homopolyubiquinations can target the substrate proteins to proteasomes, Lys48-linked polyubiquitination is preferred over the Lys63 polyubiquitination. On the other hand, mono-ubiquitination or Lys63-linked polyubiquitination may function in proteasome-independent cellular processes such as DNA repair [17], endocytosis [18], inflammation [19], and lysosomal degradation [20]. Additionally, mixed branching polyubiquitination by Lys48 and Lys63 was found to be involved in the amplification of NFKB signaling [21]. In autophagy, Lys63 polyubiquitin tags are recognized by the cargo proteins p62/SQSTM1 and NBR1 through their ubiquitin-binding domains [22], which may interact with the ubiquitin-like proteins ATG8 (LC3B) and ATG12 and facilitate degradation of ubiquitin-like and ubiquitinated proteins by autophagy [23]. However, p62/SQSTM1 has roles in both degradation pathways. When the polyubiquitin chain of the target protein binds to the ubiquitin-associated domain of p62/SQSTM1, the substrate is carried to the proteasome for degradation. 

In this study, we report that OTA stimulates transient autophagic activity during early time points after exposure, which subsides however with prolonged OTA treatment in HK-2 cells. OTA exposure increases cell death in WT MEFs but not in autophagy-halted *Atg5^-/-^* cells, suggesting that autophagy has a pro-death effect on OTA-induced cytotoxicity. Mechanistically, OTA exposure decreases global ubiquitinated protein levels by increasing proteasomal activity of chymotrypsin-, caspase-, and trypsin-like catalytic sites of purified and cellular 26S but not 20S proteasome. Despite the fact that OTA is able to directly activate protein degradation by the 26S proteasome in vitro systems, autophagy appears to be required for UPS activation in the cellular context. OTA-induced protein degradation includes the reduction of phosphatases that critically regulate PI3K/AKT and MAPK/ERK1-2 signaling. Our results therefore suggest that the combination of increased autophagic and UPS activity may cause sustained activation of PI3K/AKT and MAPK/ERK1-2 pathways through the deregulation of phosphatases, VHR/DUSP3, DUSP4, and PHLPP.

## 2. Results

### 2.1. OTA Promotes Autophagic Activity in HK-2 Cells

**Figure 1:**
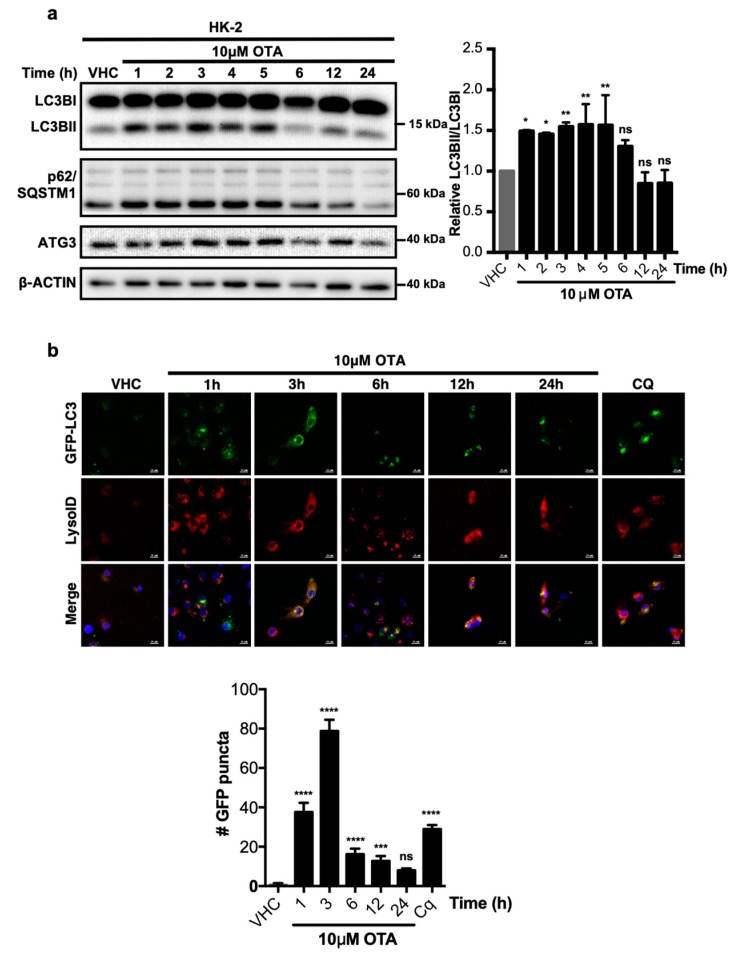
Ochratoxin A (OTA) activates autophagy as an early response in human kidney 2 (HK-2) cells. (**a**) HK-2 cells were treated with 0.1% *v*/*v* Et-OH (VHC) for 24 h or OTA (10 µM) for indicated time periods. LC3B, p62/SQSTM1, and ATG3 protein levels were detected with specific antibodies. β-ACTIN was used as loading control. Blots are representative of three independent experiments. The images were obtained from the same or parallel blots of identical samples. (**b**) Stable HK-2 cells expressing GFP fused LC3 were treated with either OTA (10 µM) for 1, 3, 6, 12, and 24 h or chloroquine (CQ, 60 µM) for 1 h or 0.1% *v*/*v* Et-OH (VHC) for 24 h. Autophagosomes (GFP-LC3) and lysosomes (LysoID^®^) were visualized in live cells. Green signals indicate LC3 (autophagosomes) proteins, red signals indicate acidic vesicles (lysosomes), and blue signals indicate nucleus. Scale Bar: 10 μM (ns: Nonsignificant, * *p* < 0.05, ** *p* < 0.01, *** *p* < 0.001, **** *p* < 0.0001).

We have previously shown that OTA stimulates sustained activity of the PI3K/AKT and MAPK/ERK1-2 signalling pathways [7] in addition to increased levels of reactive oxygen species and superoxide, which further result in protein oxidation in different cell types and animal models [10,24,25,26,27,28,29,30,31,32,33]. Therefore, we were curious whether OTA exposure activates autophagy and/or the UPS, which are involved in cellular protein homeostasis. Based on OTA dosages and stimulus durations established in our previous study [7], HK-2 cells were treated with 10 µM OTA for up to 24 h and LC3BI to LC3BII lipidation was analyzed by Western blotting as an indicator of autophagic activity (Figure 1a). Increased LC3B-II levels could be observed at early time points (between 1 and 5 h) of OTA exposure along with modest changes in p62/SQSTM1 levels, which suggests that stimulation of HK-2 cells with 10 µM OTA promotes an early autophagic response. During later time points, between 6 and 24 h of continuous OTA exposure, LC3B-II reverted to pre-stimulus levels, while p62/SQSTM1 levels were even lower at 24 h than in vehicle-treated control cells. Thus, OTA induces rapid and transient autophagic activity in HK-2 cells. 

To further confirm OTA-induced autophagic activity by direct visualization of autophagosome formation, HK-2 cells were generated that stably express green fluorescent protein fused to the LC3B protein (GFP-LC3) and acidic vesicle formation was simultaneously detected by LysoID^®^ staining (Figure 1b). Typically, LC3B joins autophagosomes and remains there until degradation of substrate and adaptor proteins by lysosomal engagement. While the GFP-LC3 fusion protein was diffusely distributed in the cytoplasm of vehicle treated HK-2 cells, the number of distinct GFP-LC3 puncta, indicative of active autophagosomes [34], increased during the first 3 h of OTA exposure. Similar to LC3B lipidation (Figure 1a), the number of autophagosomes decreased between 6 to 24 h of continuous OTA treatments and was not significantly different from vehicle-treated control cells at 24 h (Figure 1b). Critically, acidic vesicles detected by LysoID^®^ were co-localized with GFP-LC3 puncta, indicating engagement of autophagosomes with lysosomes. 

In order to examine the effect of OTA on autophagic flux, we blocked the degradation of autophagosome content with chloroquine (CQ; Figure 1b) and autophagic flux was determined as previously described [35]. OTA treatment alone moderately increased LC3BII levels over the first 6 h of treatment as before, however, simultaneous treatment of cells with OTA and CQ further increased LC3BII levels (Figure S1b). Thus, OTA treatment promotes an increase in autophagic flux over the first 6 h of treatment (Figure S1c), which may be caused by increased autophagosome formation and delivery of autophagic content to lysosomes. However, autophagic flux in OTA/CQ-treated cells was similar at 12 h and even lower at 24 h than in cells treated with CQ alone. Thus, a combination of different experimental measures to determine autophagosome formation and activity all demonstrate that autophagy is activated transiently and reverts to base levels after 6 h of sustained OTA exposure. 

### 2.2. OTA-Induced Autophagic Activity Has a Pro-Death Effect in MEF Cells 

**Figure 2:**
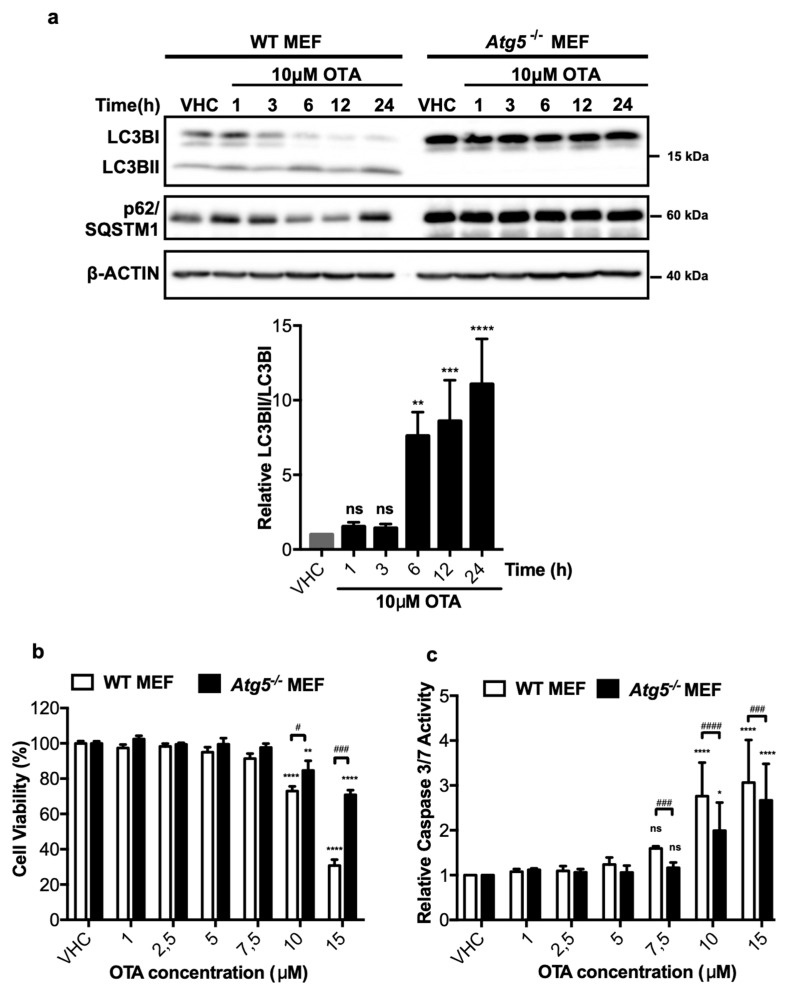
OTA activates autophagy in mouse embryonic fibroblast (MEF) cells, and wild-type (WT) MEF cells are more prone to death and apoptosis than autophagy-deficient *Atg5^-/-^* MEF cells. (**a**) MEF cells were treated with 0.1% *v*/*v* Et-OH (VHC) for 24 h or OTA (10 µM) for indicated time periods. LC3B and p62/SQSTM1 protein levels were detected with specific antibodies. β-ACTIN was used as loading control. Blots are representative of three independent experiments. The images were obtained from the same or parallel blots of identical samples. (**b**) Cell viability and (**c**) apoptotic responses were observed in WT and *Atg5^-/-^* MEF cells upon OTA exposure. MEF cells were treated with OTA at indicated concentrations or vehicle (VHC) (0.1% *v*/*v* Et-OH) for 24 h. XTT Cell proliferation kit II and caspase 3/7 assays were used to investigate cell viability and apoptotic response, respectively. Cell viability was expressed as the percent cell viability relative to the control (100%). Caspase 3/7 activity was expressed as relative to VHC control (fold change). (ns: Nonsignificant, * *p* < 0.05, ** *p* < 0.01, *** *p* < 0.001, **** *p* < 0.0001 *in group comparisons with VHC; ns: Nonsignificant, # *p* < 0.05, ### *p* < 0.001, #### *p* < 0.0001, # comparison of groups (WT and *Atg5^-/-^* MEF)).

In order to understand the possible involvement of autophagy in OTA-dependent cytotoxicity and/or carcinogenicity, we used previously established *Atg5* knock-out mouse embryonic fibroblasts (*Atg5^-/-^* MEFs) [36]. Wild-type (WT) and *Atg5^-/-^* MEF cells were treated with 10 μM OTA for up to 24 h and the extent of LC3BI to LC3BII lipidation was detected by Western blot analysis. In WT MEF cells, LC3B-II levels increased starting from the first h of OTA treatment and remained high throughout the treatment until 24 h (Figure 2a). On the other hand, p62/SQSTM1, which was also induced initially by OTA treatment, decreased between 6 and 12 h but increased again at 24 h of OTA exposure (Figure 2a). As expected for autophagy-deficient cells, lipidation of LC3BI producing LC3BII was not observed and p62/SQSTM1 levels did not change under OTA exposure (Figure 2a). 

To test the contribution of autophagy to OTA-induced cytotoxicity, WT and *Atg5^-/-^* MEF cells were exposed to increasing doses of OTA (1, 2.5, 5, 7.5, 10, and 15 μM) for 24 h and the viability of the cells was detected by XTT assays. High concentrations of OTA, 10 and 15 μM, significantly decreased the cell viability in WT MEFs to 73 ± 5.4% and 30 ± 6.6% of the viability of vehicle-treated control cells, respectively (Figure 2b). In contrast, the viability of *Atg5^-/-^* MEF cells was higher and decreased only to 70 ± 5.2% in the presence of 15 μM OTA, whereas only a modest reduction (15 ± 9.6%) was observed for the viability of cells treated with 10 μM OTA (Figure 2b). Thus, WT MEF cells are more sensitive to OTA-induced cytotoxicity compared to autophagy-deficient cells. 

To better understand the nature of the differential sensitivity of WT and *Atg5^-/-^* MEF cells, caspase 3/7 assays were performed, which revealed that OTA promotes comparable levels of apoptosis in WT and *Atg5^-/-^* MEFs at 10 and 15 μM concentrations, respectively (Figure 2c). The results demonstrate that *Atg5^-/-^* MEF cells are significantly less susceptible to OTA-induced apoptosis compared to their wild type counterparts (Figure 2c).

### 2.3. OTA Promotes Proteasomal Degradation As a Late Response in HK-2 and MEF Cells

**Figure 3:**
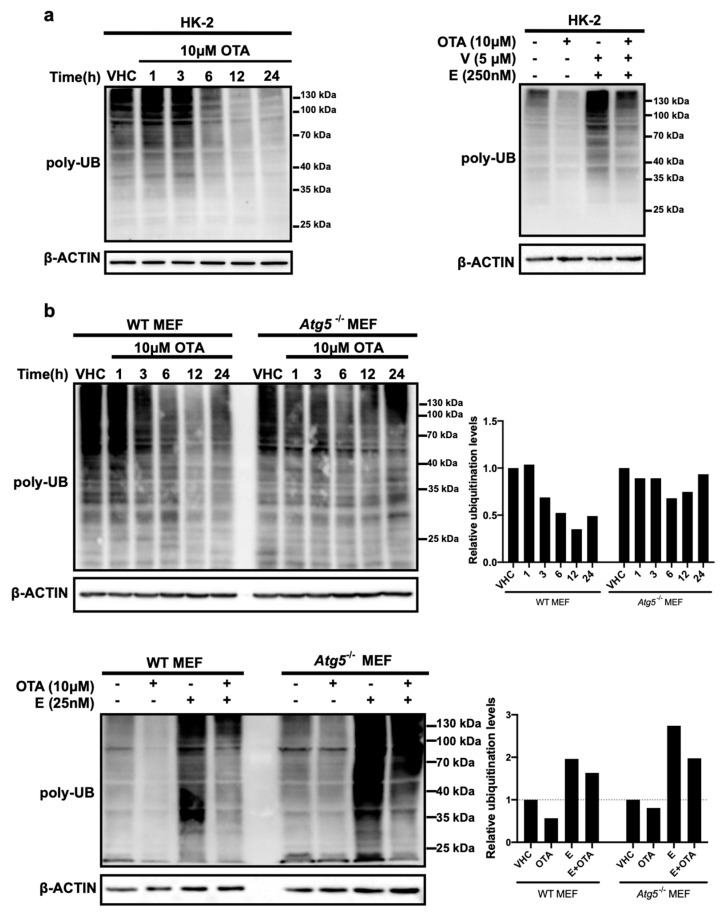
OTA activates ubiquitin-proteasome system (UPS) in HK-2 and MEF cells in further time points. (**a**) HK-2 and (**b**) MEF cells were treated with vehicle (VHC) (0.1% *v*/*v* Et-OH) for 24 h or OTA (10 µM) alone (left panel) or in combination with proteasome inhibitors, Epoxomicin (E) (250 nM for HK-2 cells and 25 nM for MEF cells) and VR23 (V) (5 µM for only HK-2 cells) for indicated time periods (right panel). Ubiquitin antibody was used to detect ubiquitinated proteins. β-ACTIN was used as loading control. Signal densities of polyubiquitinated proteins were measured and quantified relative to the vehicle treated control groups for WT and *Atg5^-/-^* MEF cells separately. Blots are representative of three independent experiments. The images were obtained from the same or parallel blots of identical samples.

Next, we examined the effects of OTA on the second major proteolytic pathway, the UPS, by determining levels of global ubiquitinated proteins in OTA- and vehicle-treated control cells (Figure 3). We reasoned that alterations in the amounts of ubiquitin conjugates may indicate the involvement of OTA in the regulation of ubiquitin-proteasome pathway. To do so, HK-2 and MEF cells (WT and *Atg5^-/-^*) were treated with 10 µM OTA for 1, 3, 6, 12, and 24 h and ubiquitinated protein levels were detected by Western blot analysis. We observed that the level of total ubiquitinated protein decreased over time in OTA treated HK-2 cells and was particularly low after 12 and 24 h (Figure 3a). In order to validate and observe the possible effect of OTA on the status of Atg5 protein beforehand, Western blot analysis was performed using extracts from WT and *Atg5^-/-^* MEF cells treated with different concentrations of OTA for 24 h. The result shows that the amount of Atg5 does not change significantly by different concentrations of OTA and it is not present in A*tg5^-/-^* MEF cells as expected (Figure S2). Remarkably, similar to HK-2 cells, OTA stimulation had a reductive effect on protein ubiquitination in WT but not in *Atg5^-/-^* MEF cells in which the levels of ubiquitinated proteins remained high even after 24 h of OTA treatment (Figure 3b). 

Decreased levels of ubiquitinated protein could arise from decreased ubiquitination by ubiquitin ligases or increased breakdown of ubiquitinated proteins by proteasomes. To discriminate between these possibilities, we treated HK-2 cells and MEFs with 10 µM OTA or a combination of OTA and the proteasome inhibitors epoxomicin and VR23 for 24 h (Figure 3a,b). The presence of proteasome inhibitors noticeably elevated the levels of ubiquitinated protein compared to vehicle control and OTA alone conditions suggesting that OTA, to some degree, promotes proteasomal degradation. Interestingly, the level of ubiquitinated proteins was marginally affected in *Atg5^-/-^* MEFs both in the absence and presence of proteasome inhibitors, suggesting that autophagy is required for OTA-induced proteasome activation. 

**Figure 4:**
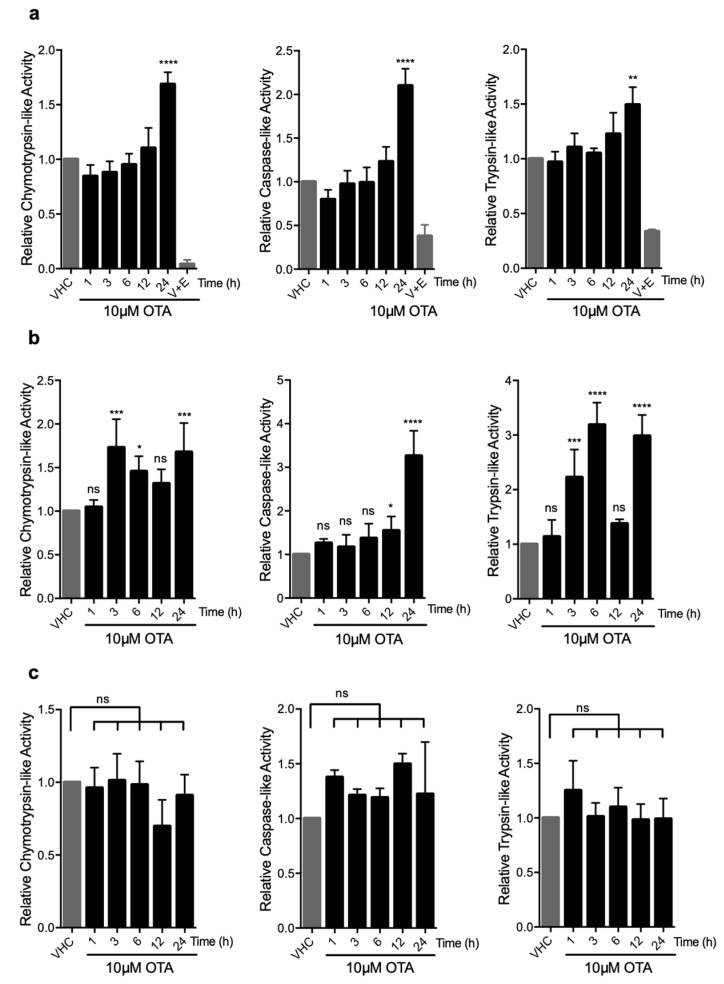
OTA elevates the enzymatic activities in 26S proteasome in HK-2 and WT MEF cells but not in autophagy-deficient *Atg5^-/-^* MEF cells. (**a**) HK-2 cells, (**b**) WT MEF cells, and **(c)** *Atg5^-/-^* MEF cells were treated with 10 µM OTA for indicated time periods or vehicle (0.1% *v*/*v* Et-OH) for 24 h. HK-2 cells were additionally treated with 10 µM OTA and proteasome inhibitors (250 nM Epoxomicin (E) and 5 µM VR23 (V) for 24 h. Lysates containing 26S proteasome were extracted from the cells and proteasome activities were measured by using Proteasome-Glo^®^ assay (Promega, USA). Suc-LLVY-Glo, Z-nLPnLD-Glo, and Z-LRR-Glo substrates were used to measure chymotrypsin-, trypsin-, and caspase-like activities, respectively. The results were normalized to PSMB5, PSMB6, and PSMB7 protein levels for chymotrypsin-, caspase-, and trypsin-like activities, respectively and expressed as relative to vehicle (VHC) control (ns: Nonsignificant, * *p* < 0.05, ** *p* < 0.01, *** *p* < 0.001, **** *p* < 0.0001).

To further investigate if OTA has a direct stimulatory effect on the proteasome and if so, which of the three enzymatic activities (trypsin-, chymotrypsin-, or caspase-like) is activated by OTA treatment, we used luminogenic substrates that are specific to each enzymic activity. Total protein lysates of vehicle- and OTA-treated HK-2 and MEF cells were admixed with specific substrates and the luminescence signals corresponding to 20S and 26S proteasome activities were analyzed (Figure 4). We observed that all three proteolytic activities increased over time and peaked at 24 h (chymotrypsin-like: 1.7 (±0.1)-fold, caspase-like: 2.1 (±0.2)-fold, and trypsin-like: 1.4 (±0.2)-fold) compared to vehicle-treated control groups in HK-2 cells (Figure 4a). The increase in activity could be blocked successfully by simultaneous application of proteasome inhibitors, demonstrating the stimulatory effect of OTA on UPS (Figure 4a). Similar results were obtained in WT MEF cells in which chymotrypsin-, caspase-, and trypsin-like activities peaked to 1.6 (±0.3)-, 3.3 (±0.6)-, and 3.0 (±0.4) -fold at 24 h of OTA exposure, respectively (Figure 4b). Interestingly, there was no significant change in any of the proteolytic activities in OTA-treated autophagy-deficient *Atg5^-/-^* MEF cells compared to WT control MEFs and HK-2 cells (Figure 4c). It, thus, appears that induction of autophagy is a prerequisite for the activation of the UPS and degradation of ubiquitinated proteins in OTA exposed cells.

### 2.4. OTA-Induced UPS Activity is Not Related to the Levels of the Proteasome Subunits

**Figure 5:**
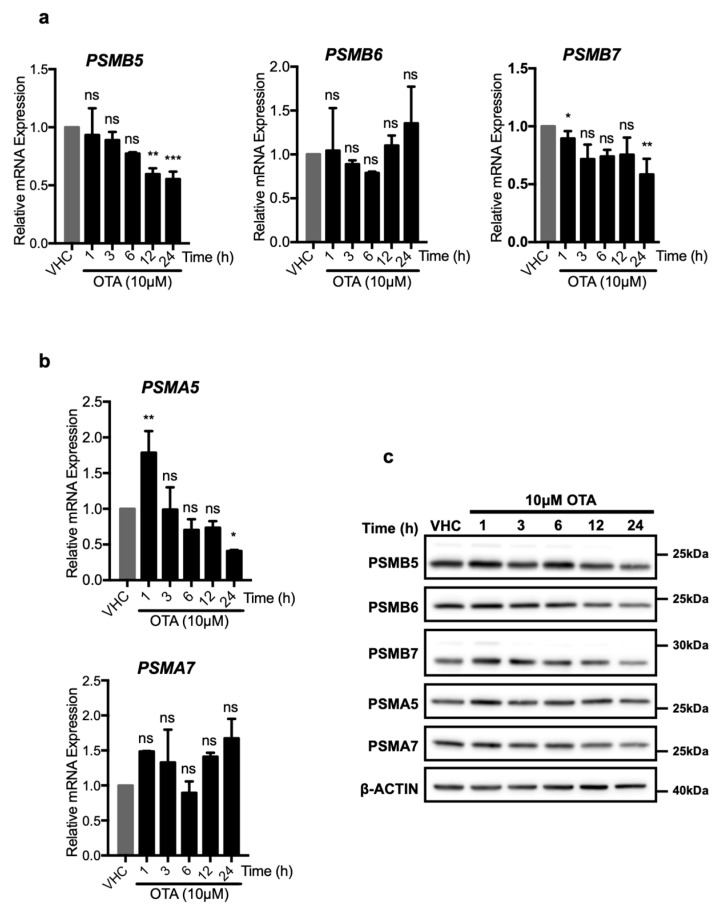
OTA decreases the expressions of the proteasome subunits both at mRNA and protein levels. HK-2 cells were treated with OTA (10 µM) for 1, 3, 6, 12, and 24 h or vehicle (0.1% *v*/*v* Et-OH) for 24 h. Changes on the mRNA levels of (**a**) PSMB5, PSMB6, and PSMB7 forming proteasome β subunit and (**b**) PSMA5 and PSMA7 forming proteasome α subunit were analyzed with RT-qPCR with the primer sets given in Table S1. mRNA levels were shown relative to β-ACTIN and vehicle (VHC) control (2^−ΔΔCt^ method). For the statistical analysis one-way ANOVA and Bonferroni (as post-hoc) tests were used. (**c**) HK-2 cells were treated with OTA (10 µM) for 1, 3, 6, 12, and 24 h or vehicle (VHC) (0.1% *v*/*v* Et-OH) for 24 h. Changes on the protein levels of the 26S proteasome subunits were detected with Western blot analysis. β subunit of 26S proteasome was analyzed with antibodies specific to PSMB5, PSMB6, and PSMB7 proteins, α subunit was analyzed with PSMA5 and PSMA7 specific antibodies. β-ACTIN antibody was used as loading control. Blots are representative of three independent experiments. The images were obtained from the same or parallel blots of identical samples (ns: Nonsignificant, * *p* < 0.05, ** *p* < 0.01, *** *p* < 0.001, **** *p* < 0.0001).

OTA may enhance proteasome activity in two ways, which are not necessarily mutually exclusive to each other: By increasing expressions of proteasome proteins and therefore the number of available proteasomes, or by directly altering the enzymatic activity of existing proteasomes. To further investigate these possibilities, HK-2 cells were treated with OTA and the expressions of proteasome proteins were analyzed at mRNA and protein levels (Figure 5). The PSMB5, PSMB6, PSMB7 proteins are responsible for the chymotrypsin-, caspase-, and trypsin-like activities of the β subunit, respectively, while PSMA5 and PSMA7 are components of the proteasomal α subunit. Of those, the *PSMB5* and *PSMB7* mRNA levels decreased significantly, especially at 24 h, whereas *PSMB6* mRNA levels did not differ over the 24 h of OTA exposure (Figure 5). mRNA level of *PSMA5* significantly increased to 1.78-fold of control cells during the first h but decreased rapidly during subsequent time points of the OTA treatment, while *PSMA7* mRNA levels were not affected. 

Similarly, PSMA5 protein level increased slightly during the first hour (Figure 5c) and further treatment with OTA decreased PSMA5 protein levels at 24 h. Moreover, no significant increase in PSMA7 protein levels were observed in OTA exposed HK-2 cells (Figure 5c). Furthermore, it was observed that, while PSMB7 and PSMA5 protein levels increased to a small degree in the earlier time points, PSMB7 decreased to the level below the control group in 12 and 24 h. PSMB5, PSMB6, and PSMA7 protein levels did not change at the earlier time points while they decreased in 24 h of OTA exposure of HK 2 cells (Figure 5c).

With the exception of PSMA5, which increased early after OTA treatment, none of the α and β catalytic subunits of the 26S proteasome was elevated at mRNA or protein levels at 12 and 24 h, the time points at which increased proteasome activity was observed. On the contrary, the levels of some of the proteins (PSMB5, PSMB6, and PSMB7) even decreased significantly at these times. The results, thus, suggest that the increased proteasome activity caused by OTA exposure was caused by increased catalytic activity of existing proteasome complexes.

### 2.5. OTA Directly Increases the Proteolytic Activities of the 26S But Not the 20S Proteasome

**Figure 6:**
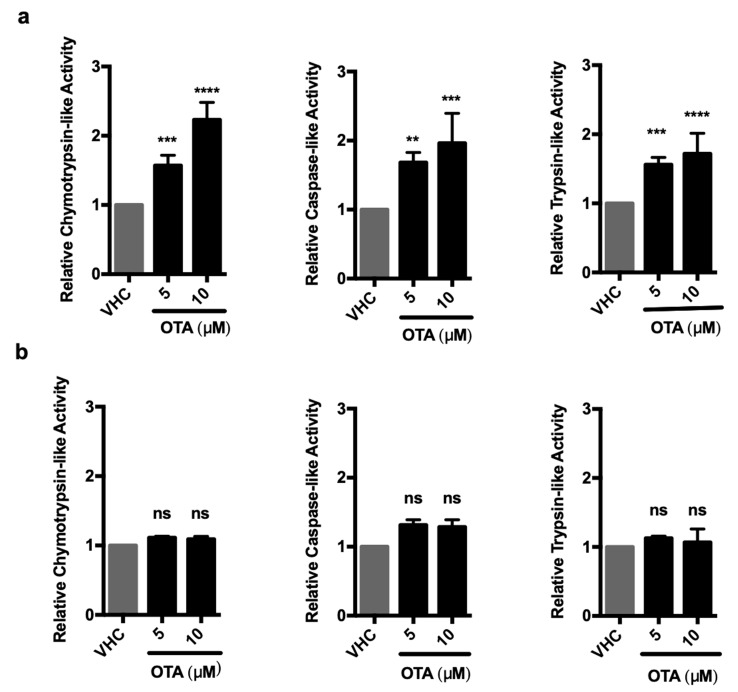
OTA directly activates 26S pure proteasome but not 20S pure proteasome. (**a**) 26S and (**b**) 20S (0.3 ng) pure proteasome complexes were treated with 5 or 10 µM OTA or vehicle (VHC) (0.2% *v*/*v* Et-OH) in 10 mM HEPES buffer (pH 7.6) for 1 h at 37 °C. OTA-treated 26S and 20S proteasome complexes were mixed with specific luminogenic substrates (Suc-LLVY-Glo, Z-nLPnLD-Glo, and Z-LRR-Glo for chymotrypsin-, caspase-, and trypsin-like activities, respectively) in the presence of 250 μM ATP for 26S proteasome and luminogenic signals were detected. Results were shown relative to the vehicle (VHC) control (ns: Nonsignificant, ** *p* < 0.01, *** *p* < 0.001, **** *p* < 0.0001).

In order to investigate the direct or any differential effect of OTA on the activities of 20S and 26S proteasomes, in vitro peptidase activities of purified proteasomes were measured in the presence of OTA (Figure 6). Commercially available purified 20S and 26S proteasomes were treated with OTA and their activities were measured using the Proteasome-Glo^®^ Assay. OTA at 5 μΜ concentration increased chymotrypsin-like activity by 1.57 (±0.15)-fold, caspase-like activity by 1.68 (±0.15)-fold, and trypsin-like activity by 1.56 (±0.1)-fold relative to the vehicle control treatment in 26S proteasomes. The effect was dose-dependent and even higher activities (chymotrypsin-like: 2.23 (±0.25)-fold, caspase-like: 1.96 (±0.43)-fold, and trypsin-like: 1.71(±0.30)-fold) were observed when 10 µM OTA was used (Figure 6a). However, the activity of the 20S proteasome did not change significantly upon 5 or 10 μΜ OTA treatments (Figure 6b). These results, therefore, indicate that OTA specifically increases the activity of the 26S proteasome.

### 2.6. OTA Stimulates Degradation of the Exogenous UPS Substrate, Ubiquitinated-Green Fluorescence Protein (Ub-GFP)

Since OTA specifically enhanced the enzymatic activity of the 26S proteasome in cell lysates and purified proteasomes, we wanted to further confirm these observations in intact cells by examining the levels of those exogenously expressed ubiquitinated-GFP proteins, which are specific UPS substrates. For this analysis, Ub-GFP chimeras with differential sensitivity to proteasomal degradation were used: Ub^G76V^-GFP and Ub^R^-GFP are highly susceptible to degradation by 26S proteasome, while Ub^M^-GFP cannot be processed and is stable [37]. Moreover, Ub^G76V^-GFP and Ub^R^-GFP are degraded by proteasome through ubiquitin fusion degradation and N-end rule degradation, respectively, and both forms were used to examine the effects of OTA on UPS activity. As HK-2 cells are difficult to transfect, HeLa cells were transiently transfected with Ub-GFP reporter constructs and treated with 10 µM OTA alone or in combination with the proteasome inhibitors VR23 and epoxomicin and GFP signals were visualized and quantified by flow cytometric analysis.

When stimulated with OTA, Ub^G76V^-GFP and Ub^R^-GFP signals decreased by half compared to their levels in vehicle-treated control cells (Figure S4a,b). As expected, the proteasome inhibitors epoxomicin and VR23 increased both the number of cells expressing GFP and the mean intensity value of GFP signals compared to vehicle controls in both Ub^G76V^-GFP and Ub^R^-GFP expressing cells (Figure S3a,b). Additionally, when cells were treated with OTA and proteasome inhibitors, the intensity of GFP signals and the numbers of the cells expressing GFP increased in comparison to OTA-only treatment conditions (Figure S4a,b) confirming that OTA decreases ubiquitinated-GFP levels through the activation of proteasomes. As expected, OTA and proteasome inhibitors did not cause any significant change in the GFP levels in cells expressing the stable Ub^M^-GFP variant (Figure S4c). The results show that degradation of ubiquitinated protein substrates by 26S proteasomes in response to OTA stimulation also in intact cells and is not an artifact that is specific to purified proteasomes. 

### 2.7. OTA Decreases the Half-Lives of the Natural Substrate Proteins of UPS; MCL1, KEAP1, and IKBα

MCL1 [38], KEAP1 [39], and IKBα [40] are natural substrates of the UPS, which mediate regulation of the expressions of important survival and apoptotic proteins. To provide further evidence on the stimulatory effect of OTA on proteasomes, we examined the half-lives of these endogenous UPS substrates in the presence of OTA. HK-2 cells were treated with cycloheximide (CHX), which blocks translation, in the presence or absence of OTA (Figure S5a). This comparison is informative in the sense that any observed change in protein levels would be due to altered protein half-lives rather than changes in the translation rate of these proteins. We observed that MCL1, KEAP1, and IKBα diminished at a steeper rate when cells were exposed to CHX and OTA compared to the CHX alone condition, supporting the notion that OTA promotes faster protein degradation (Figure S5a). Moreover, when cells were treated with the proteasome inhibitors epoxomicin and VR23, the levels of MCL1, KEAP1, and IKBα increased in the presence of OTA. Thus, epoxomicin and VR23 reversed the effect of OTA on protein degradation (Figure S5b), confirming that the observed degradation was the result of increased proteasomal activity.

### 2.8. OTA Facilitates Degradation of Phosphatases Involved in PI3K/AKT and MAPK/ERK1-2 Pathways Through Activation of Autophagy and UPS

We have previously shown that OTA activates the PI3K/AKT and MAPK/ERK1-2 pathways by analyzing the phosphorylation status of AKT (Ser307 and Ser473) and ERK1-2 (Thr202/204) proteins and that increased activity is sustained for up to 48 h of OTA exposure [7]. Phosphatases, PHLPP (SCOP), PTEN, DUSP4, and VHR/DUSP3, are responsible for dephosphorylation of AKT (Ser307 and Ser473) and ERK1-2 (Thr202/204) and they are natural substrates of autophagic and proteasomal degradation. Since OTA decreases global ubiquitinated proteins (Figure 3) we wanted to investigate if sustained activation of PI3K/AKT and MAPK/ERK1-2 pathways is caused by decrease in the levels of the phosphatases. Therefore, we examined the levels of the phosphatases PHLPP (SCOP), PTEN, DUSP4, and VHR/DUSP3 by Western blot analysis in OTA-treated HK-2, and WT and *Atg5^-/-^* MEF cells. Of those, PHLPP (SCOP) and PTEN are involved in de-phosphorylation of AKT (Ser473) and DUSP4, VHR/DUSP3 are responsible for the dephosphorylation of p-ERK1-2 (Thr202/204). 

**Figure 7:**
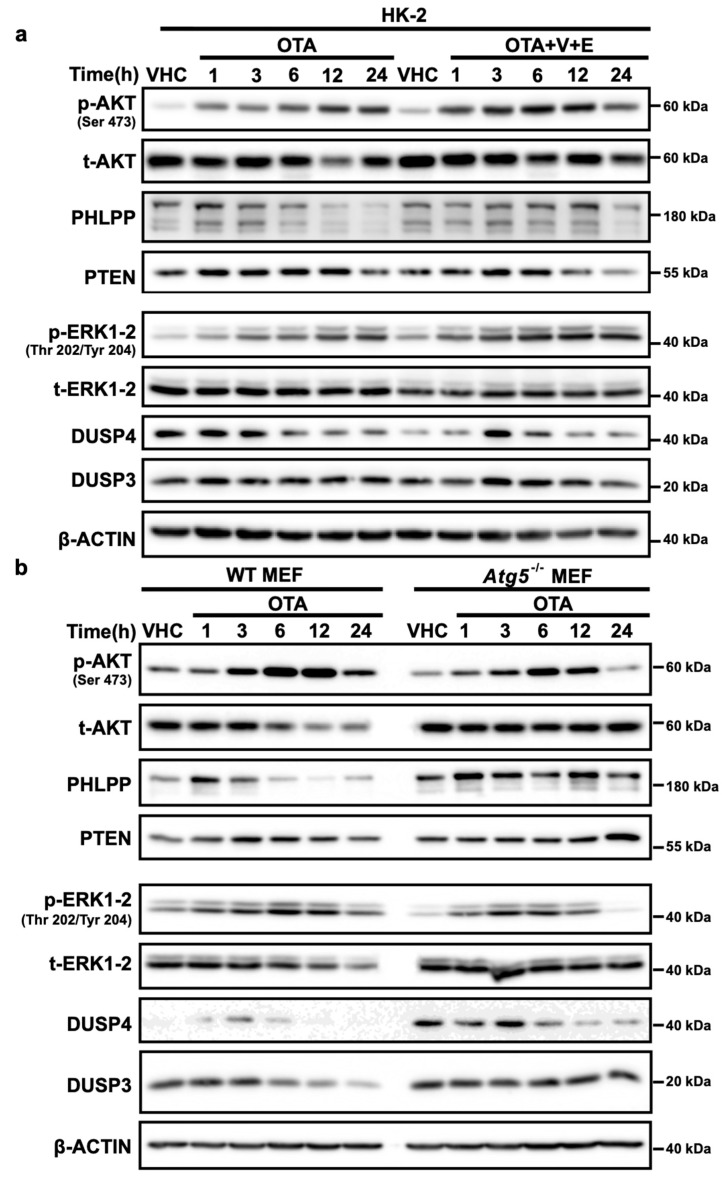
OTA facilitates the degradation of phosphatases involved in PI3K/AKT and MAPK/ERK1-2 pathways. (**a**) HK-2, (**b**) WT, and *Atg5^-/-^* MEF cells were treated with 10 µM OTA for 1, 3, 6, 12, and 24 h or vehicle (VHC) (0.1% *v*/*v* Et-OH) for 24 h. (**a**) Additionally, HK-2 cells were treated with Epoxomicin (E) (250 nM) and VR23 (V) (5 µM) simultaneously with OTA for 1, 3, 6, 12, and 24 h. p-AKT, t-AKT, PHLPP, PTEN, p-ERK1-2, t-ERK1-2, VHR/DUSP3, and DUSP4 levels were detected with specific antibodies by Western blot analysis. β-ACTIN was used as loading control. Blots are representative of three independent experiments. The images were obtained from the same or parallel blots of identical samples.

We observed that PHLPP, DUSP4 and VHR/DUSP3 levels decreased significantly over time upon 10 μΜ OTA exposure in both HK-2 and WT MEF cells (Figure 7). Total PTEN levels did not change significantly but the levels of the phosphorylated form of PTEN was shown to be decreased [7] upon OTA exposure as its activation would be regulated not only by degradation but also through phosphorylation. Interestingly, the basal levels of PHLPP (SCOP), PTEN, DUSP4, and VHR/DUSP3 were found to be higher in autophagy-deficient *Atg5^-/-^* MEF cells than in WT MEF cells. Similarly, OTA did not facilitate the decrease of PHLPP (SCOP), PTEN, DUSP4, and VHR/DUSP3 levels in *Atg5^-/-^* MEF cells (Figure 7b) suggesting that induction of the autophagic process is necessary. Importantly, phosphorylation of both AKT and ERK decreased to the basal levels (vehicle control) confirming that OTA did not promote sustained activation of PI3K/AKT and MAPK/ERK1-2 in autophagy-deficient *Atg5^-/-^* MEF cells. 

Furthermore, to examine the effect of OTA-induced UPS activity on the levels of the phosphatases, HK-2 cells were treated with OTA in combination with proteasome inhibitors and the levels of the PHLPP, DUSP4, and VHR/DUSP3 proteins were detected by Western blot analysis. We observed that the presence of proteasome inhibitors prevented degradation of the phosphatases during the first 12 h of OTA treatment but that the levels of PHLPP, DUSP4, and VHR/DUSP3 proteins decreased drastically by 24 h (Figure 7a) where autophagy was further activated by the presence of proteasome inhibitors (Figure S6). These results strongly indicate that autophagy has to be functional and regulate the levels of the phosphatases alone or together with UPS in sustained activation of PI3K/AKT and MAPK/ERK1-2 pathways in the presence of OTA.

## 3. Discussion

Thus far, OTA has been proposed to have both genotoxic and non-genotoxic effects [41]. OTA was associated with Balkan Endemic Nephropathy (BEN) [42]. It is believed to cause kidney injury and even tumor formation upon prolonged exposure. Some claim that OTA is a genotoxic substance that causes the formation of DNA adducts and genomic instability [6] and other researchers argue that it has epigenetic effects where signaling pathways are deregulated resulting in suppression or promotion of apoptosis and cell cycle progression [8,9]. 

We had previously demonstrated that OTA causes sustained activation of PI3K/AKT and MAPK/ERK1-2 pathways in HK-2 cell line [7]. These two pathways were also shown to work in opposite directions in which PI3K/AKT pathway supports survival response whereas MAPK/ERK1-2 activation promotes apoptosis in the cells [43]. Moreover, it was shown that OTA activates PI3K/AKT pathway in part through receptor tyrosine kinase c-Met activation [7]. However, the upstream effector of MAPK/ERK1-2 pathway has yet to be elucidated. Upstream effectors aside, it is largely unclear how these signaling pathways remain constitutively active under OTA exposure.

Autophagy and UPS-mediated protein degradation play important roles in the regulation of a number of signaling pathways and removal of undesired protein aggregates which could be toxic otherwise [44]. Therefore, these two proteolytic pathways have pivotal roles in the cellular livelihood. Our results suggest that OTA provokes apoptotic cellular death through activation of autophagy. These findings are consistent with the study by Shen et al. [11] reporting that mitochondrial receptor protein Nix mediates autophagic degradation of mitochondria (mitophagy), which then mediates and promotes cellular death in OTA toxicity. However, it is not clear if apoptosis or lysosomal cellular death is regulated directly by mitochondria itself or by the upstream effectors whose expression profiles are changed by Nix in survival and/or apoptotic signaling pathways in their study [11].

Inappropriate proteasome activity can derail cellular signaling cascades by modulating the levels of kinases and phosphatases involved in survival and apoptotic pathways. In addition, defunctionalized or less active proteasomes could hamper removal of toxic protein aggregates that result in the development of neurological disorders like spinocerebellar ataxia, amyotrophic lateral sclerosis (ALS), Alzheimer, and Huntington. Therefore, there is an ever-increasing effort in the field of drug discovery aiming at identification of small molecules that target proteasomes. There are several small molecules identified that enhance proteasomal activity [45,46,47]. However, IU1 is the only small molecule that enhances proteasomal degradation through inhibiting the de-ubiquitination of the polyubiquitinated substrates by the USP14 subunit [48]. In the current study, we demonstrated that OTA enhances the activity of 26S proteasome by acting directly on the proteasomes similar to IU1 but the mechanism of how OTA directly augments the activity of proteasome still needs to be clarified and is under investigation.

For a long time, researchers thought that autophagy and UPS are independent proteolytic pathways. However, these views have been challenged by the recent studies showing that they are highly connected to each other [49,50,51,52,53,54]. The activation of each system depends on several factors such as ATP and amino acid needs, protein quality, and aggregate formations [55]. Depending on the factors, each pathway may be activated alone or together. While UPS mostly degrades short-lived proteins, long-lived proteins are degraded by both autophagy and UPS [56]. It was previously shown that both autophagy and UPS can be regulated by the same upstream effectors as mTOR (mammalian Target of Rapamycin). When the mTOR pathway is inhibited, cell growth slows down and both autophagy and UPS are activated where only the long-lived proteins are degraded in order to replenish amino acid source [57]. Moreover, UPS activity can be regulated via the cAMP-PKA pathway through the phosphorylation of the proteasome component Rpn6/PSMD11 of 19S regulatory particle leading to UPS-dependent degradation of mostly short-lived proteins [47]. 

At this crossroad, p62/SQSTM1 draws much attention as it is one of the cargo receptor proteins affecting both autophagy and UPS. It has ubiquitin-binding domain which interacts with ubiquitinated substrate proteins and directs them to degradation via UPS or autophagy. Additionally, p62/SQSTM1 has a protein binding domain which modulates the accumulation of p62/SQSTM1-ubiquitinated protein aggregates in the cells [58]. The levels of p62/SQSTM1 in the cells effect the decision whether proteins undergo UPS- or autophagy-mediated degradation [59]. Proteasome inhibitors activate autophagy as a compensatory pathway for the degradation of ubiquitinated short-lived and long-lived proteins interacting with p62/SQSTM1 [60]. However, when autophagy is genetically blocked, short-lived proteins do not go through proteasomal degradation and p62/SQSTM1 accumulates simultaneously with ubiquitinated proteins [56]. 

In our current study, it was shown that autophagy is activated in the earlier time periods of OTA treatment but autophagic activity is decreased in later time points when the proteasomal degradation is enhanced. It was previously reported that inhibition of UPS activates autophagy by which degradation of ubiquitinated proteins continues; however, when autophagy is blocked ubiquitinated proteins complexed with p62/SQSTM1 inhibit the function of UPS by obstructing the substrate reception [56]. 

In the present study, it was also demonstrated that ubiquitinated protein levels and p62/SQSTM1 levels do not decrease and 26S proteasome activity does not increase in the lysates obtained from autophagy deficient Atg5^-/-^ MEF cells exposed to OTA at later time points. These two results suggest that OTA does not promote proteasomal degradation in autophagy-deficient Atg5^-/-^ MEF cells. Therefore, we concluded that autophagy has to be functional for the activation of UPS upon OTA exposure in the cellular context.

Interestingly, our in vitro proteasome assays demonstrated that OTA directly enhanced the activity of pure 26S proteasomes (Figure 6). In addition, OTA did not increase the expressions of proteasome components such as PSMB5, PSMB6, and PSMB7 neither at mRNA nor protein levels (Figure 5). From these results, we speculate that when autophagy is blocked in the cells, ubiquitinated proteins cannot be handed over to the catalytic region of the 26S proteasome even though p62/SQSTM1 and ubiquitinated proteins are accumulated in the cells. Therefore, UPS activity is not promoted in *Atg5^-/-^* MEF cells by OTA (Figure 3b, Figure 4c). Similarly, Korolchuk et al. previously reported that when autophagy is blocked, p62/SQSTM1 and ubiquitinated proteins accumulate in the cells preventing the delivery of ubiquitinated proteins destined for the proteasomal degradation [61].

**Figure 8:**
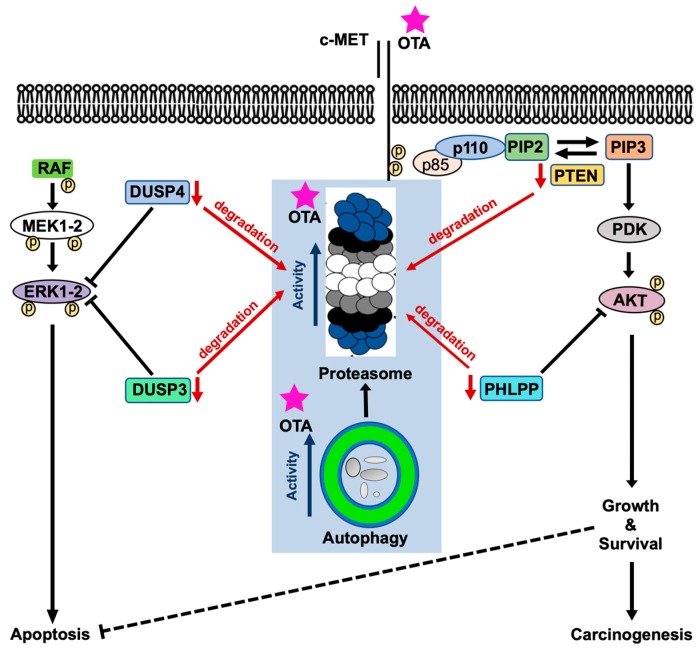
Proposed model of the mode of action of OTA on proteolytic, MAPK/ERK1-2, and PI3K/AKT pathways. Red arrows indicate degradation, blue arrows indicate activities, and black arrows and lines indicate signaling route.

Phosphatases are responsible for the de-phosphorylation of the particular proteins involved in cellular signaling pathways. As mentioned earlier, PI3K/AKT and MAPK/ERK1-2 pathways are activated upon OTA exposure in HK-2 cells. Activation of these pathways is sustained up to 48 h of OTA treatment [7]. Therefore, we wondered if this continuous activation resulted from decreased phosphatase levels. As expected, OTA decreased the levels of PHLPP, VHR/DUSP3, and DUSP4 in a time-dependent manner in HK-2 and WT MEF cells, which may well explain sustained activation of PI3K/AKT and MAPK/ERK1-2 pathways (Figure 7 and Figure 8). In *Atg5^-/-^* MEF cells, however, OTA could not reduce the levels of the phosphatases throughout the treatments where the phosphorylation of AKT and ERK1-2 decreased to vehicle control conditions in 24-h of OTA treatment. Moreover, when HK-2 cells were treated with OTA and the proteasome inhibitors, the levels of the phosphatases were stable up to 12 h but decreased conspicuously within 24 h as proteasome inhibitors activated autophagy (Figure S6). In general, PHLPP [62] and DUSP4/MKP2 [63] are polyubiquitinated and regulated by UPS during cellular metabolism. Although the dynamics of the short-lived proteins like phosphatases are regulated predominantly by UPS, it is also well-known that the inhibition of autophagy causes impaired degradation of short-lived proteins by UPS [61]. Therefore, it is biologically plausible to speculate that OTA-induced autophagy and UPS together decrease the levels of phosphatases involved in the deactivation of PI3K/AKT and MAPK/ERK1-2 pathways leading to sustained activation of these pathways (Figure 8).

## 4. Conclusions

This is the first report showing that OTA sequentially activates autophagy and UPS. We showed that autophagy sensitizes the cells to OTA-induced apoptotic death. Moreover, OTA directly induces peptidase activities in 26S proteasome which requires functional autophagy in the cellular context. Enhanced activities of autophagy and UPS by OTA decrease the levels of the phosphatases DUSP3, DUSP4, and PHLPP involved in MAPK/ERK1-2 and PI3K/AKT signaling pathways. Finally, we believe that the low levels of these negative regulators are the reasons for the sustained activities of MAPK/ERK1-2 and PI3K/AKT signaling pathways under OTA exposure [7].

## 5. Materials and Methods 

### 5.1. Cell Culture

Human kidney 2 (HK-2) cells were purchased from American Type Culture Collection (ATCC) (Manassas, USA). Immortalized wild-type and Atg5^-/-^ mouse embryonic fibroblasts (MEF) were kindly provided by Prof. Devrim Gözüaçık, Sabancı University, Istanbul, Turkey [36,64]. HeLa cells were kindly provided by Dr. Cemalettin Bekpen from Max Planck Institute for Evolutionary Biology, Germany.

HK-2, HeLa, and MEF cells were cultured in DMEM/F12 (Pan Biotechnologies, Aidenbach, Bavaria, Germany) supplemented with 10% fetal bovine serum (FBS Gibco, Life Technologies, USA) and Penicillin (100 U/mL)-Streptomycin (100 µg/mL) (Gibco, Life Technologies, USA) at 37 °C, 5% CO_2_, and humidified atmosphere. They were grown in 60, 100, or 150 mm cell culture dishes and were sub-cultured every other day. The FBS content of medium was decreased to 5% during the chemical and toxin treatment conditions.

### 5.2. Stable Cell Line

In order to eliminate the stress factors in transient transfection processes, HK-2 cells that stably express GFP-LC3 protein were generated. For stable transfection, cells were transfected with pBabe_GFP-LC3 (pBABEpuro GFP-LC3 was a gift from Jayanta Debnath (Adgene plasmid # 22405)) by using Lipofectamine 2000 (Invitrogen Life Technologies, Carlsbad, CA, USA) and cells carrying the plasmid were subjected to selection with 10 μg/mL Puromycin for three weeks.

### 5.3. Chemicals and Treatments

OTA (Product number: O1877-25mg), autophagy inhibitor chloroquine (Product number: C6628) and cycloheximide (Product number: C7698) were purchased from Sigma-Aldrich (Darmstadt, Germany). Proteasome inhibitors; VR23 (Product number: S7933) was purchased from Sellekchem (Houston, TX, USA), Epoxomicin (Product number: A2606) was purchased from Apexbio (Boston, MA, USA). All the other chemicals were purchased from Sigma-Aldrich (Darmstadt, Germany) unless it is indicated otherwise.

Cells were treated with OTA for indicated time periods and concentrations specific to the experimental set up. Application doses of OTA were selected based on our group’s previous studies [7]. Stock solution of OTA and chloroquine were prepared in ethanol (Et-OH). Proteasome inhibitors, VR23, and Epoxomicin, were dissolved in DMSO. Control cells were treated with vehicles, 0.1% *v*/*v* Et-OH and/or 0.1% *v*/*v* DMSO. Inhibitor treatments were performed before OTA administration to the cells.

### 5.4. Cell Viability Assay

Proliferation of the cells grown in a DMEM-F12 medium was analyzed with XTT Cell proliferation kit II (Roche, Switzerland). Cell viability was measured upon chemotoxic response. MEF cells (7.5–10 × 10^3^) were seeded into the 96-well plates and treated with 1, 2.5, 5, 7.5, 10, and 15 µM OTA for 24 h. XTT reagent and electron coupling reagent were mixed and directly added to the cells in the medium. After 3-h incubation at 37 °C, absorbance values were measured at 490 and 655 nm wavelengths by microplate reader (Bio-Rad, Hercules, CA, USA). The absorbance values are correlated with the metabolic activity of the cells.

### 5.5. Protein Extraction and Western Blot Analysis

After toxin and chemical treatments, cells were harvested in RIPA (150 mM NaCl_2_, 1% NP40, 0.5% sodium deoxycolate, 0.1% SDS, 50 mM Tris, pH 7.4) supplied with Phos-STOP phosphatase inhibitor cocktail (Roche Applied Sciences, Basel, Switzerland), and one tablet Complete EDTA-free protease inhibitor cocktail (Roche Applied Sciences, Basel, Switzerland). Concentrations of the protein lysates were quantified by DC Protein Assay (Bio-Rad Laboratories, Hercules, CA, USA) as the manufacturer recommended. Total protein lysates (15–25 µg) were run on 10% or 12% acrylamide-bis-acrylamide (37.5:1) gel. Before loading, samples were mixed with Laemmli buffer (200 mM Tris-HCl pH 6.8, 8% SDS, 40% Glycerol, 4% β-Mercaptoethanol, 50 mM EDTA, 0,08% Bromophenol Blue) and denatured at 95 °C for 5 min. Proteins were run at 100 and 150 V on stacking and resolving gel, respectively. After running, proteins were transferred to the PVDF membrane (Thermo Scientific, Waltham, MA, USA) and membranes were blocked in blocking solution (5% skimmed milk prepared in TBS-T [50 mM Tris-HCl pH 7.4, 150 mM NaCl, % 0.1 Tween–20]) for 1 h at room temperature (RT) on an orbital shaker. Some of the membranes were cut into pieces to incubate with multiple antibodies. Next, membranes were incubated with indicated first antibodies in 5% BSA in TBS-T (1:1000) overnight at 4 °C. Then, membranes were incubated with HRP-linked second antibody (anti-Rabbit #7074 or anti-Mouse #7076 IgG) (Cell Signaling Technologies, Danvers, MA, USA) in blocking solution for 1 h at RT. Then, membranes were incubated in HRP substrate-enhancer solution (Advansta, WesternBright ECL HRP Substrate, San Jose, CA, USA) for about 1 min. Finally, protein bands were visualized with the chemiluminescence visualization system (GBox Chemi, Syngene, Cambridge, UK) and were analyzed by Genetools software from the same company and ImageJ (NIH, Bethesda, MD, USA) image analysis software. Primary antibodies used for protein detection are as follows: Ubiquitin #sc-8017; PHLPP/SCOP #sc-390129; PSMA7 #sc-58417 were ordered from Santa Cruz Biotechnologies Inc., (Dallas, TX USA). p-p44/p42 (T202/Y204 p-ERK1/2) #4370p; p44/42 (ERK1/2) #4695s; pAKT (S473) #4060s; AKT #4691s; DUSP3/VHR #4752; DUSP4/MKP2 #5149; PTEN #9188; p-PTEN(Ser380) #9551; KEAP1 #8047s; MCL1#4572; IKBα #4814; PSMB5 #12919s; PSMB6 #13267s; PSMB7 #13207s; PSMA5 #2457s; ATG5 #8540p; β-ACTIN #8457L were ordered from Cell Signaling Technologies (Danvers, MA, USA) and p62/SQSTM1 #P0067 and LC3B #L7543 were ordered from Sigma-Aldrich (Darmstadt, Germany). β-ACTIN was used as loading control and for normalization purposes to express relative quantity of chemiluminescence signals where it is necessary.

### 5.6. Detection of Autophagy and Acidic Vesicles upon OTA Treatment in HK-2 Cell Line

Autophagy induction was investigated by examining LC3B-I to LC3B-II conversion by Western blotting and visualizing LC3B puncta formation in stable HK-2 cells expressing GFP-LC3 fusion protein. Stable GFP-LC3 expressing HK-2 cells were treated with 10 µM OTA for 1, 3, 6, 12, and 24 h. As vehicle control, cells were treated with 0.1% *v*/*v* Et-OH for 24 h. In order to detect acidic vesicles, Lyso-ID Red Detection Kit (Enzo Life Sciences, Farmingdale, NY, USA) was used as recommended by the manufacturer. Briefly, cells were stained with detection solution; red detection dye for the acidic vesicles, and Hoechst to stain the nuclei. Stained cells were visualized with Leica TCS SP8 confocal microscope (Wetzlar, Germany).

### 5.7. Measurement of Proteasome Activity

Proteasome-Glo 3 substrate system (Promega, Madison, WI, USA) was used for the detection of proteasome activity. The assay was performed as described in the manual provided by the manufacturer with a few modifications. Briefly, 6 × 10^6^ cells were seeded into 150 mm culture plate. Next day, cells were treated with vehicle (0.1% *v*/*v* Et-OH) for 24 h or OTA (10 µM) for 1, 3, 6, 12, and 24 h or OTA (10 µM), Epoxomicin (250 nM), and VR23 (5 µM) for 24 h. Cell lysates containing 26S proteasome, were obtained from treated cells. For the luminometric detection; substrates specific to each proteolytic activity were prepared as recommended by the manufacturer in the protocol were mixed with the cell lysates and shaken at 300–500 rpm for 30 s. After shaking they were incubated at RT for about 1 h and luminescence measurements were performed by using Fluoroskan Ascent FL Microplate Fluorometer and Luminometer (Thermo Scientific, Waltham, MA, USA). Equal amount of total protein (50 μg) was used in all experimental groups for the comparison. To investigate the direct effects of OTA on the proteasome, commercially purchased pure 20S and 26S proteasomes (0.3 ng) were treated with OTA (5 and 10 µM) for 1 h and activities of OTA exposed pure proteasomes were measured as described above.

### 5.8. Detection of the Expression Levels of the Components of 26S Proteasome by RT-qPCR

HK-2 cells (1 × 10^6^) were seeded in 60 mm culture dishes. Next day cells were treated with vehicle (0.1% Et-OH) for 24 h or 10 µM OTA for 1, 3, 6, 12, and 24 h in a DMEM-F12 culture medium containing 5% FBS. After the treatments, cells were washed with PBS and the RNA extraction kit (Zymo, Irvine, CA, USA) was used as described by the manufacturer. Briefly, cells were lysed with Tri-reagent (MRC gene, USA) and RNAs were captured on columns. Any contaminant DNA was removed with DNase treatment. Extracted RNA was quantified with NanoDrop 2000 (Thermo Scientific, Waltham, MA, USA) and stored in −80 °C freezers until use. cDNA was synthesized by iScript cDNA synthesis kit (Bio-Rad, Hercules, CA, USA) as described by the manufacturer. Briefly, 1 μg total RNA was mixed with oligo (dT), 5x reaction mix containing random hexamer primers and reverse transcriptase enzyme in 20 μL reaction volume. cDNA was synthesized at 46 °C for 20 min and reverse transcriptase was inactivated by incubation at 95 °C for 1 min. cDNA samples were diluted 1:5 and used as a template in further qPCR analysis. In order to check the expression levels of PSMB5, PSMB6, PSMB7, PSMA5, and PSMA7 genes at the mRNA level upon OTA exposure, primers specific to each gene (Table S1) and β-ACTIN (as control) were designed at exon-exon junctions. cDNA samples were used as template and amplified with DNA SensiFast SYBR Mastermix (Bioline, London, UK). PSMB5, PSMB6, PSMB7, PSMA5, and PSMA7 mRNA expressions in OTA-treated cells for different time points were shown relative to the expression levels in vehicle treated-control group by using the 2^−ΔΔCt^ method [65].

### 5.9. Transient Transfection of the HeLa Cells with Ub-GFP Plasmids, Visualization under Fluorescence Microscope, and Flow Cytometric Analysis

HeLa (5 × 10^5^) cells were seeded in 6-well culture plates. Next day, cells were transfected with Ub^G76V^-GFP (Addgene plasmid # 11941), Ub^R^-GFP (Addgene plasmid # 11939), and Ub^M^-GFP (Addgene plasmid # 11938) reporter constructs (valuable gifts from Nico Dantuma) [37] by using Lipofectamine 2000 (Invitrogen Life Technologies, Carlsbad, CA, USA) as the manufacturer recommended. Next day cells were treated with vehicle (0.1% *v*/*v* Et-OH) for 24 h or OTA (10 µM) alone or in combination with proteasome inhibitors, VR23 (5 µM) and Epoxomicin (250 nM). After 24 h, GFP signal was visualized with inverted fluorescence microscope (Eclipse TS100, Nikon, Tokyo, Japan) and quantitatively analyzed by flow cytometry using C6 Acuri, BD (Becton Dickinson, Franklin Lakes, NJ, USA). 

### 5.10. Statistical Analysis

Statistical analysis was performed with GraphPad Prism version 7.0a for Mac OS X. The data was shown as mean ± SD and for comparisons, one-way ANOVA and Bonferroni (as post-hoc test) tests were used. For all of the tests, significance criterion was set to *p* < 0.05. All experiments were repeated independently, at least three times.

## Supplementary Materials

The following are available online at https://www.mdpi.com/2072-6651/11/11/615/s1, Figure S1: OTA induces autophagosome formation and their delivery to autophagolysosomes in the first 6-h treatments; Figure S2: OTA does not affect ATG5 protein expression in WT MEF cells; Table S1: Primers used in RT-qPCR; Figure S3: OTA elevates the enzymatic activities in 26S proteasome in HK-2 and WT MEF cells but not in autophagy-deficient *Atg5^-/-^* MEF cells; Figure S4: OTA facilitates the degradation of ubiquitinated GFP substrates; Figure S5: OTA facilitates the degradation of the endogenous substrates (IKBα, KEAP-1, and MCL1) of UPS; Figure S6: Proteasome inhibitors further activates OTA-induced autophagy.
