# Supplementary material for: Ochratoxin A Sequentially Activates Autophagy and the Ubiquitin-Proteasome System
Source: Toxins (Basel). 2019 Oct 24;11(11):615. doi: 10.3390/toxins11110615 (PMC6891609; doi:10.3390/toxins11110615)
Supplement: Supplementary file 1 [file toxins-11-00615-s001.pdf]

Supplementary Materials: Ochratoxin A Sequentially Activates Autophagy and the Ubiquitin-Proteasome System

Hafize Aysin Akpinar, Hilal Kahraman and Ibrahim Yaman

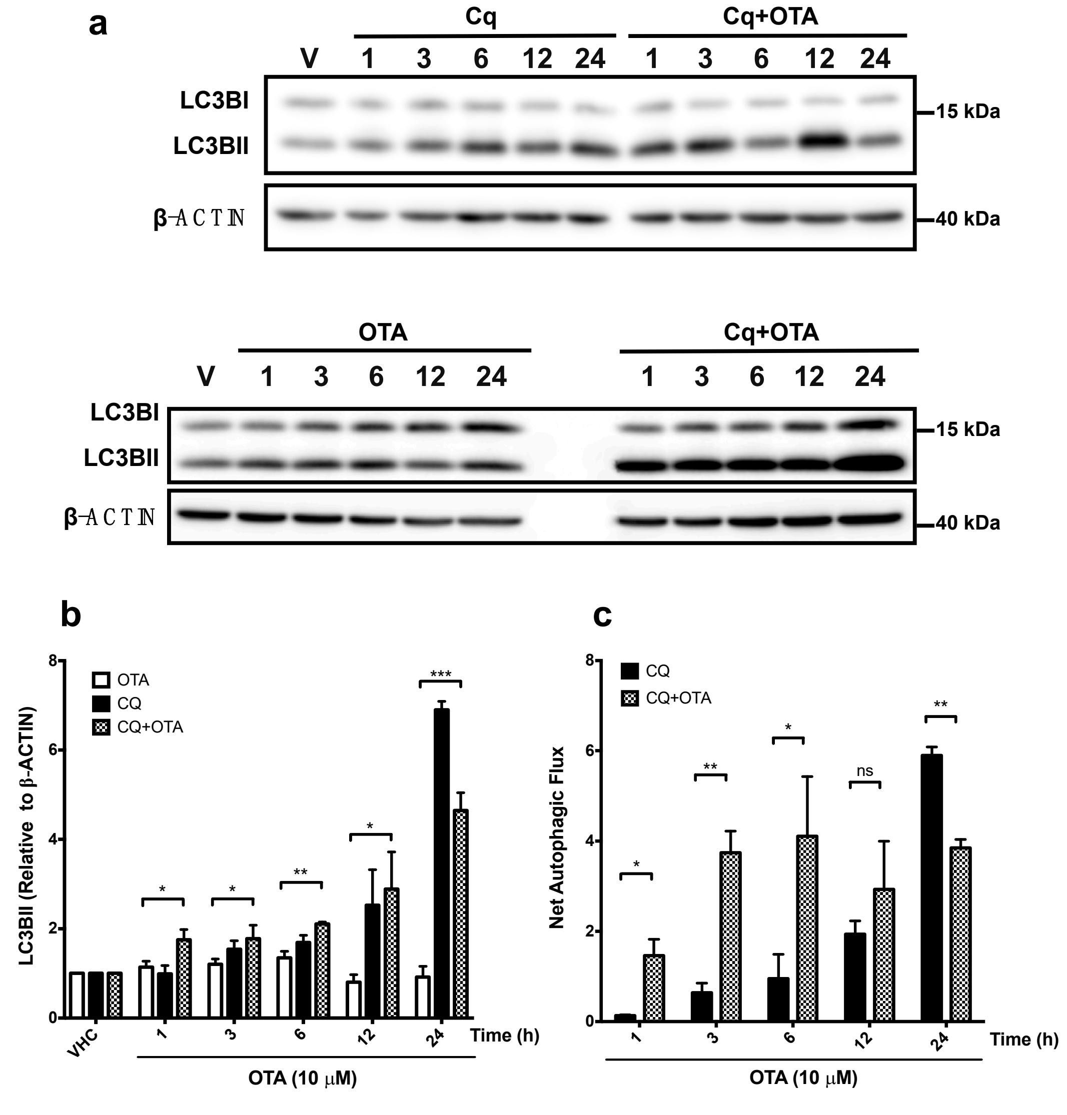

Figure S1. Ochratoxin A (OTA) induces autophagosome formation and their delivery to autophagolysosomes in the first 6 h treatments. (**a**) HK-2 cells were treated with chloroquine (CQ) (20 μM) alone or in combination with OTA (10 μM) for 1, 3, 6, 12 and 24 h or vehicle (VHC) (0.1% *v*/*v* Et-OH) for 24 h. Blots are representative of three independent experiments. The images were obtained from the same or parallel blots of identical samples (**b**) LC3BII and β-ACTIN bands were quantified with ImageJ software, LC3BII signals (relative to β-ACTIN) were shown relative to the vehicle (VHC) control. (**c**) Autophagic flux was shown by calculating the difference in LC3BII levels between CQ or VHC treated cells (CQ group in graph) or CQ+OTA or OTA treated cells (CQ+OTA CQ group in graph) (ns: Nonsignificant, * $p < 0.05$, ** $p < 0.01$).

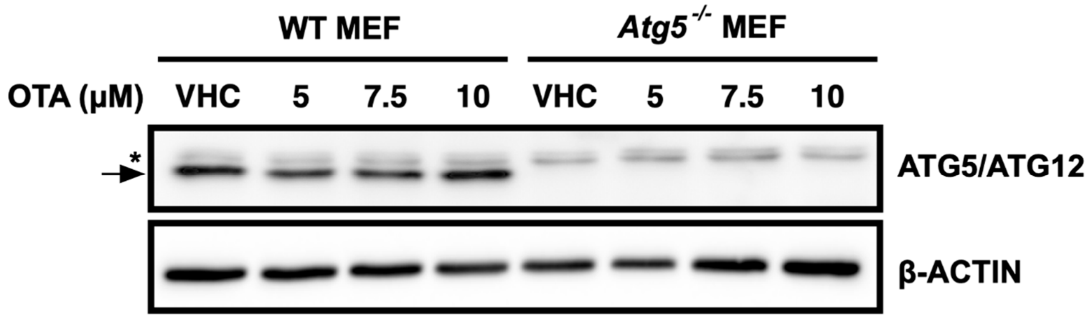

Figure S2. OTA does not affect ATG5 protein expression in wild-type mouse embryonic fibroblast (WT MEF) cells. WT and *Atg5*$^{-/-}$ MEF cells were treated with OTA (5, 7.5 and 10μM) or vehicle (VHC) (0.1% *v*/*v* Et-OH) for 24 h. ATG5 level was detected with ATG5 specific antibody by Western blot analysis. β-ACTIN was used as loading control.

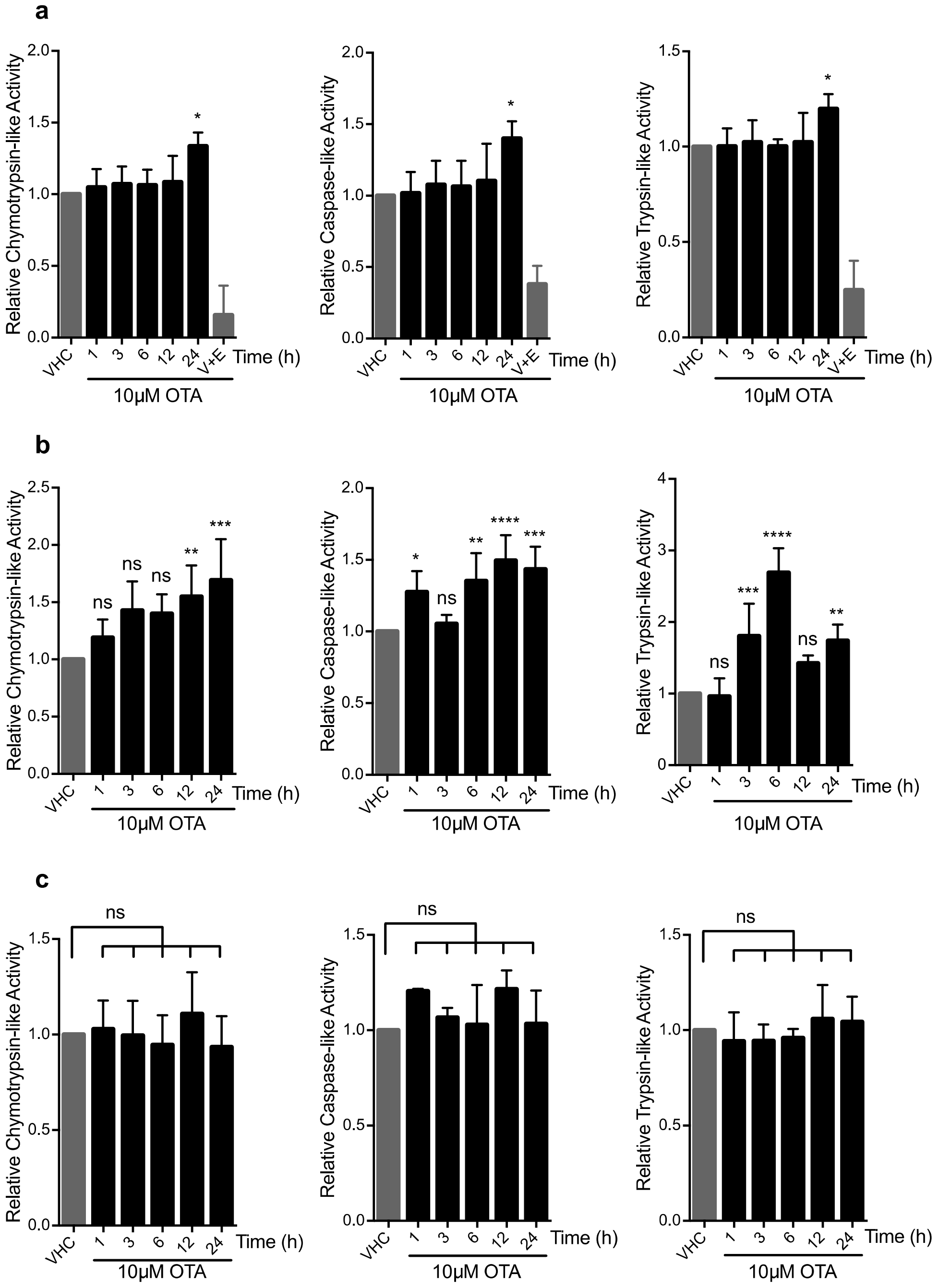

Figure S3. OTA elevates the enzymatic activities in 26S proteasome in HK-2 and WT MEF cells but not in autophagy-deficient *Atg5*$^{-/-}$ MEF cells. (**a**) HK-2 cells (**b**) WT MEF cells and (**c**) *Atg5*$^{-/-}$ MEF cells were treated with 10μM OTA for 1, 3, 6, 12, 24 h or vehicle (VHC) (0.1% Et-OH) for 24 h. Lysates containing 26S proteasome were extracted from the cells and proteasome activities were measured by using Proteasome-Glo® assay (Promega, Madison, WI, USA). Suc-LLVY-Glo, Z-nLPnLD-Glo and Z-LRR-Glo substrates were used to measure chymotrypsin-, trypsin- and caspase-like activities respectively. The results were normalized to total protein levels and expressed as relative to vehicle (VHC) control (ns: Nonsignificant, * $p < 0.05$, ** $p < 0.01$, *** $p < 0.001$, **** $p < 0.0001$).

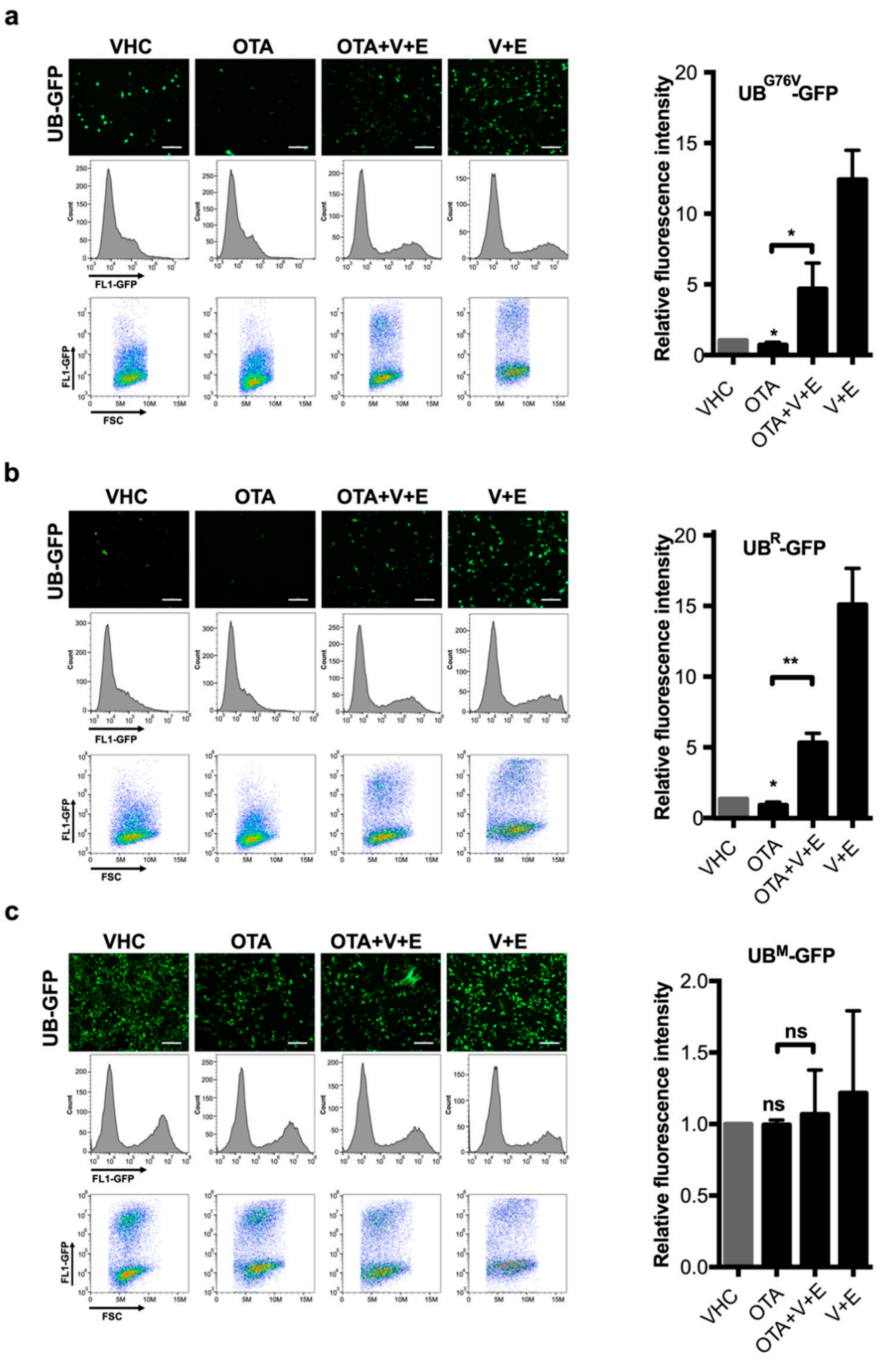

Figure S4. OTA facilitates the degradation of ubiquitinated Green Flourescent Protein (GFP) substrates. Degradation of Ub^{G76V}-GFP, Ub^{R}-GFP and Ub^{M}-GFP upon OTA exposure in the absence or presence of the proteasome inhibitors (VR23 and Epoxomicin) were investigated. HeLa cells were transiently transfected with either (**a**) Ub^{G76V}-GFP or (**b**) Ub^{R}-GFP or (**c**) Ub^{M}-GFP and treated with OTA (10μM) or vehicle (0.1% *v*/*v* Et-OH) alone or in combination with Epoxomicin (E) (250 nM) and VR23 (V) (5 μM) for 24 h. Fluorescence images were obtained with inverted fluorescence microscope (Scale bar, 100 μm) (Nikon Eclipse TS100,

Tokyo, Japan). GFP signals were quantified with C6 Acuri (BD, Franklin Lakes, NJ, USA). FL1 versus Count graphs were used to demonstrate mean values of GFP and FSC versus FL1 graphs were used to show the GFP positive and negative cell populations. Quantified GFP signals were expressed as relative fluorescence intensity to vehicle (VHC) control (ns: Nonsignificant, * $p < 0.05$, ** $p < 0.01$, **** $p < 0.0001$).

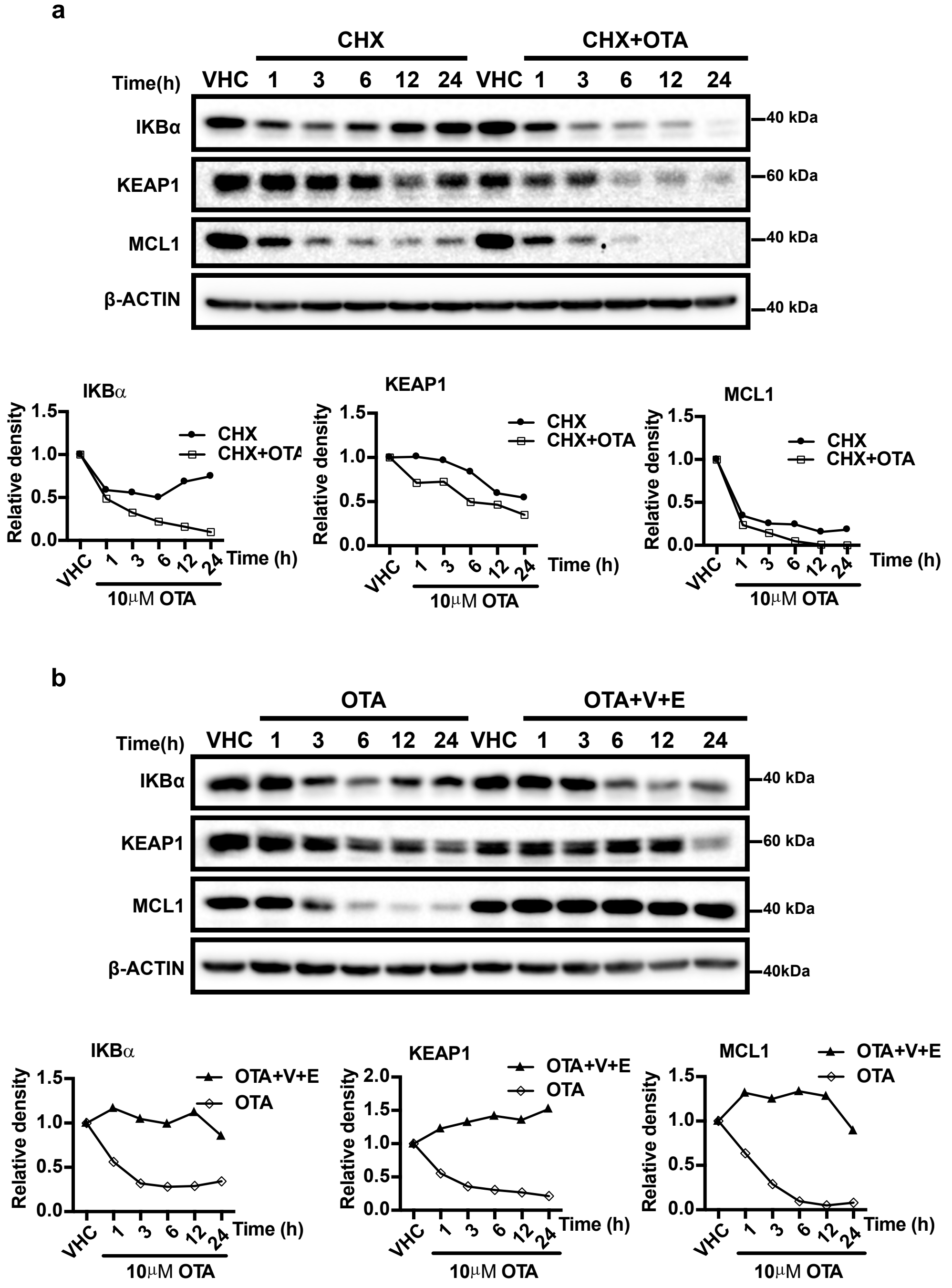

Figure S5. OTA facilitates the degradation of the endogenous substrates (IKBα, KEAP-1 and MCL1) of UPS. (**a**) HK-2 cells were treated with CHX (10 μg/ml) alone or in combination with OTA (10 μM) for 1, 3, 6, 12 and 24 h or vehicle (VHC) (0.1% *v*/*v* Et-OH) for 24 h. (**b**) HK-2 cells were treated with OTA (10 μM) alone or

in combination with for Epoxomicin (E) (250 nM) and VR23 (V) (5 μM) for 1, 3, 6, 12 and 24 h or vehicle (VHC) (0.1% *v*/*v* Et-OH) for 24 h. Western blot analysis was performed to detect IKBα, KEAP-1 and MCL1 obtained from treated cells. β-ACTIN antibody was used as loading control. Protein bands were quantified with ImageJ software, which was shown relative to the vehicle (VHC) control. Blots are representative of three independent experiments. The images were obtained from the same or parallel blots of identical samples.

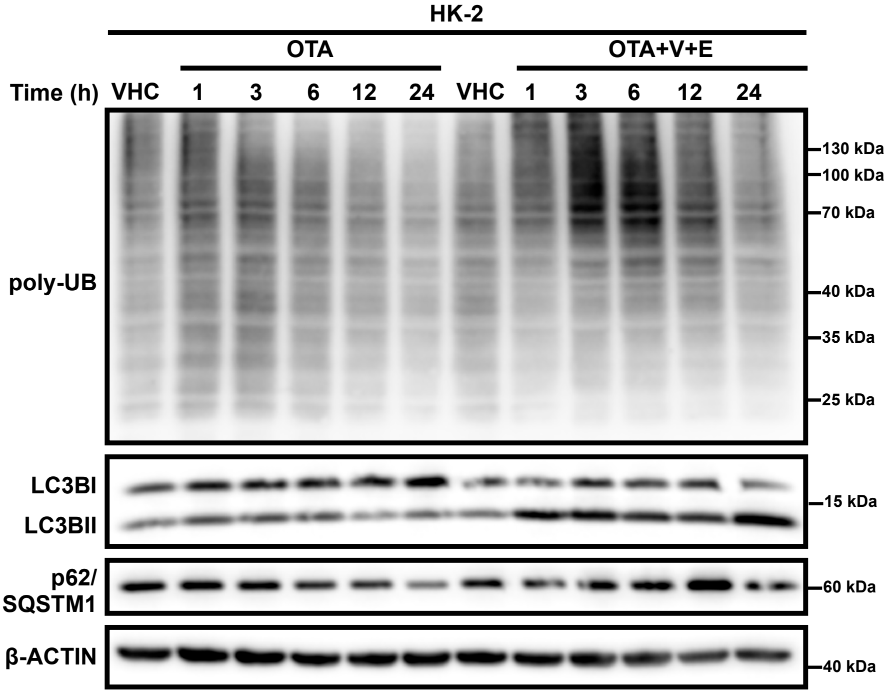

Figure S6. Proteasome inhibitors further activates OTA-induced autophagy. HK-2, cells were treated with 10 μM OTA for 1, 3, 6, 12 and 24 h or vehicle (VHC) (0.1% Et-OH) for 24 h alone or in combination with Epoxomicin (E) (250 nM) and VR23 (V) (5μM). Poly-UB, LC3B and p62/SQSTM1 levels were detected with specific antibodies by Western blot analysis. β-ACTIN was used as loading control. Blots are representative of three independent experiments. The images were obtained from the same or parallel blots of identical samples.

Table S1. Primers used in RT-qPCR.

Gene	Forward Primer	Reverse Primer
PSMB5	5′-GTGTCCCAGAAGAGCCAGGA-3′	5′-GCAATGTAAGCACCCGCTGTA-3′
PSMB6	5′-CTCCACTGGTCCACACAGCA-3′	5′-ACTGAGTACACCTGCCCTCC-3′
PSMB7	5′-TGGCCGTCTTCCCAGAGTTG-3′	5′-GGAGCCAGAACCCATGGTGA-3′
PSMA5	5′-TCCAGGTGCCATGTCTCGTC-3′	5′-CACCCTCTGAAGCAGAGCCA-3′
PSMA7	5′-CCGCTACATCGCCAGTCTGA-3′	5′-GAACTCGCGCACTGACTTGG-3′
β-ACTIN	5′-TCCTGGGCATGGAGTCCTGT-3′	5′-TCTGCTGGAAGGTGGACAGC-3′
